# Hypertension Control Status Among Patients Receiving Treatment From Selected Primary Health Centres in Puducherry: A Cohort-Analysis Approach

**DOI:** 10.7759/cureus.45042

**Published:** 2023-09-11

**Authors:** Aanchal Arora, Anurag Gola, Vignesh Loganathan, Subitha Lakshminarayanan, Sitanshu Sekhar Kar

**Affiliations:** 1 Department of Preventive & Social Medicine, Jawaharlal Institute of Postgraduate Medical Education & Research, Puducherry, IND

**Keywords:** socio-demographic factors, cohort analysis, cohort monitoring, control rate, hypertension

## Abstract

Background: High blood pressure (hypertension) is a major risk factor contributing to 60% of premature deaths caused by non-communicable diseases. In India, a mere 15% of the hypertensive population achieves optimal blood pressure control. Effective monitoring of hypertension is crucial for mitigating the morbidity and mortality associated with cardiovascular diseases.

Objective: This study employed a cohort analysis approach to determine the control status of hypertension and identify factors associated with hypertension among individuals seeking care at selected primary health centres (PHCs) in Puducherry from January 2019 to December 2022.

Methodology: We assessed treatment records of 1127 patients with hypertension registered at PHCs in both urban and rural areas between 2019 and 2022. Information on socio-demographic details and blood pressure readings was collected to assess the control status of hypertension on a quarterly and six-monthly basis. Additionally, 436 patients were interviewed to identify factors associated with uncontrolled hypertension.

Results: Control rates of hypertension varied among PHCs on a quarterly and six-monthly basis. The rural PHC achieved the highest quarterly control rate of 80% in Q4 2020, while the urban PHC had the lowest rate of 44% in Q1 2020. Similarly, the highest six-monthly control rate of 78% was observed in Q3 2019 at both rural and urban PHCs, with the lowest rate of 44% in Q1 2020 at the urban PHC.

Conclusion: Analysing data obtained from regular monitoring of hypertension control status allows healthcare providers to identify patterns, trends, and correlations. It assists providers in making informed decisions regarding treatment adjustments, medication choices, lifestyle recommendations, and policy changes. This approach is expected to improve control status for hypertension, leading to the ultimate goal of better health outcomes for patients.

## Introduction

Hypertension, or high blood pressure (BP), is defined as a systolic BP of 140 mm Hg or higher or a diastolic BP of 90 mm Hg or greater [1]. Globally over one billion people are living with hypertension [2]. According to the National Non-communicable Disease Monitoring Survey (NNMS), hypertension currently affects nearly three out of ten adults in India [3]. The prevalence of hypertension is 21.3% for females and 24% for males [4]. In Puducherry, 33% of individuals between 18 and 69 years have elevated BP [5]. In low and middle-income countries, 60% of deaths are attributed to non-communicable diseases (NCDs), highlighting hypertension as a major public health concern [6]. In India, the hypertension control rate, i.e., systolic BP and diastolic BP below 140 mmHg and 90 mmHg, respectively, is reportedly 15% [7]. Various factors such as age, general health, underlying medical issues, socio-demographic characteristics, behavioural patterns, lifestyle choices, and clinical factors contribute to the challenge of achieving adequate hypertension control rates in India [8].

A cross-sectional epidemiological survey is often used to estimate hypertension prevalence and control status. However, BP and the control status of hypertension are not one-time measurements, and they may change over time. To account for this, the World Health Organization (WHO) - HEARTS technical package recommends a cohort analysis approach to track the longitudinal control of hypertension at the level of primary healthcare centres [9]. Also, this approach can identify patients with persistently uncontrolled hypertension. The monitoring indicators recommended in the WHO-HEARTS technical package, including the longitudinal monitoring of hypertension, were adopted by the India Hypertension Control Initiative (IHCI). The IHCI 2021 report disseminated the increased registrations of people with hypertension and their control rate at health and wellness centres (HWCs), primary health centres (PHCs), hospitals, and community health centre (CHC) facilities [10].

In this study, we aim to determine the control status of hypertension among patients registered at selected PHCs in Puducherry using a cohort analysis approach and also to determine the factors associated with uncontrolled hypertension.

## Materials and methods

Study design and setting

This record-based study was conducted in two selected PHCs in the Puducherry district situated on India's south-eastern coast. The selected PHCs were the urban primary health centre (UPHC) and rural primary health centre (RPHC) of a tertiary care hospital in Puducherry, and they cater to approximately 10,000 population, each. The two PHCs were purposively selected as they are the training centres for the institute and have complete records of registered patients with hypertension. The UPHC and RPHC have 710 and 910 registered NCD patients, respectively. Monthly NCD clinics are conducted at these PHCs to provide comprehensive care for patients with NCDs. During each visit to the NCD clinic, patients undergo essential laboratory tests, including BP measurement. Additionally, medical officers, nurses, and medical social workers are actively involved in dispensing monthly medications, delivering health education, and providing counseling on lifestyle modifications, adherence to medical management, and self-care practices.

Sample size and study period

The study evaluated the treatment records of 1127 patients diagnosed with hypertension, with 622 patients registered at the UPHC and 505 patients registered at the RPHC. The records were reviewed between April 2022 and January 2023. Additionally, we employed a simple random sampling technique using Microsoft Excel to select 436 participants with hypertension attending the NCD clinic for interviews to investigate factors associated with high BP. These participants were selected from the line list available at the PHCs, covering the period from April 2022 to January 2023.

Study procedure

We extracted socio-demographic details and BP readings from the records of participants with hypertension who attended NCD clinics in selected PHCs. Then interviews were conducted among the chosen subgroup of patients using a structured questionnaire. The questionnaire aimed to collect information about different socio-demographic factors, following the acquisition of informed consent. These interviews were carried out during their scheduled follow-up visits at the NCD clinics in the PHCs.

Data analysis

**Figure 1:**
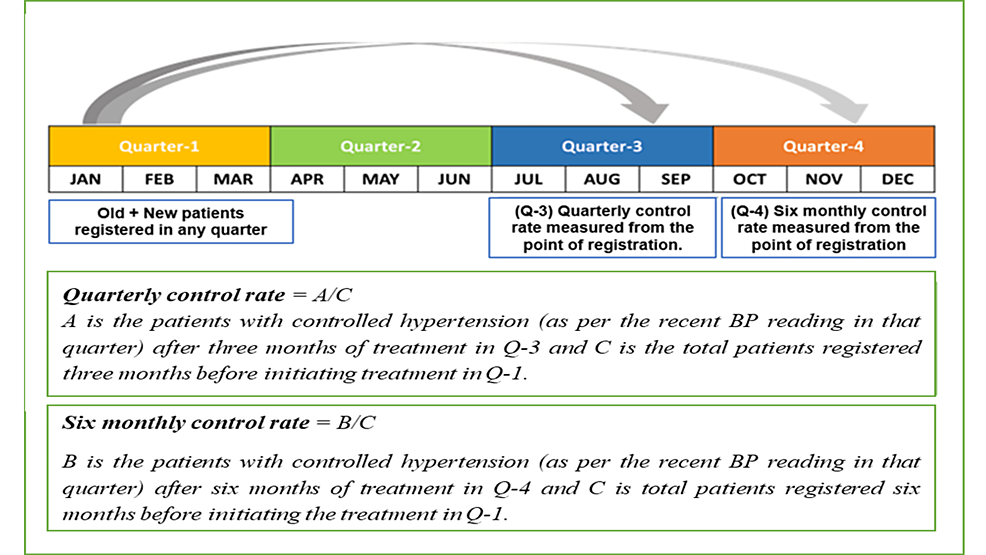
Method to calculate the quarterly and six-monthly hypertension control status

We reviewed BP readings for each month over a four-year period (January 2019 to December 2022) to determine the quarterly and six-monthly control statuses of hypertension. The control status of hypertension was defined as having a systolic BP below 140 mm Hg and a diastolic BP below 90 mm Hg during the most recent visit within the respective quarter. The control status year was divided into four quarters (Q1: January to March, Q2: April to June, Q3: July to September, and Q4: October to December) (Figure 1). The quarterly and six-monthly control rates for hypertension were calculated as the proportion of patients receiving treatment for hypertension at the health facility whose BP was controlled after three and six months of treatment, respectively [8]. Individuals with hypertension were also classified as having "adequately controlled hypertension" if their BP was under control for 10 or more months during the year. Data entry was performed using Epi-collect software (ver. 4.1.5), and data analysis was conducted using IBM SPSS Statistics for Windows, Version 23 (Released 2015; IBM Corp., Armonk, New York, United States). Continuous variables are presented as mean with standard deviation (SD) or median with interquartile Range (IQR), depending on data normality, while categorical variables are reported as frequencies with percentages. The association between socio-demographic factors and hypertension control was assessed by estimating the prevalence ratio along with 95% confidence intervals and a p-value of less than 0.05.

Ethics approval

The study protocol and other relevant documents have received approval from JIPMER Institutional Ethics Committee for Observational Studies with the reference number JIP/IEC/2022/130.

## Results

**Figure 2:**
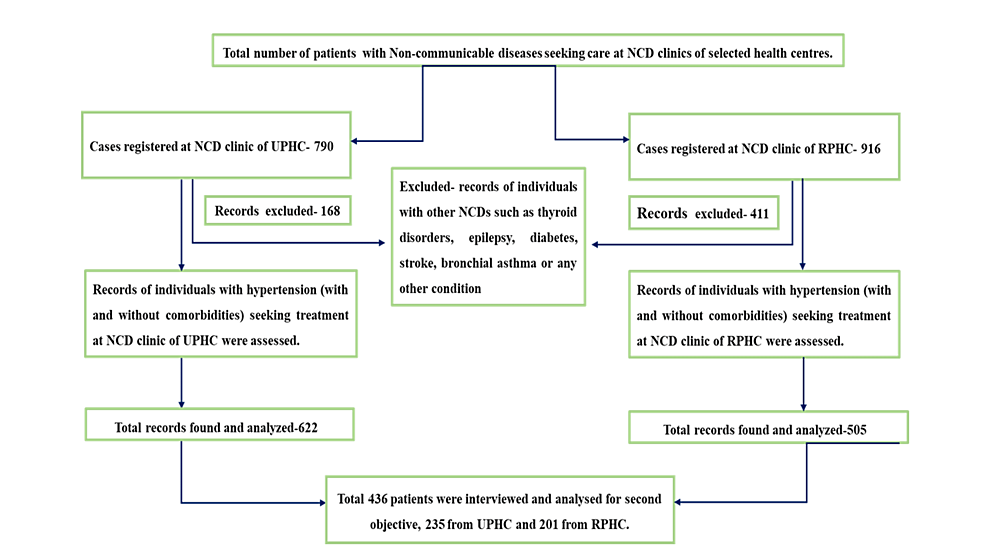
Flow diagram showing the process of selecting patient records for the study NCD: Non-communicable disease; RPHC: rural primary health centre; UPHC: urban primary health centre

The patient record selection process is depicted in Figure 2. The socio-demographic characteristics, comorbidity status, and hypertension treatment details of the included patients in the study are presented in Table 1. The follow-up proportion until Q-2 of 2022 among registered participants was 81% for the UPHC and 89% for the RPHC.

**Table 1:** Characteristics of patients with hypertension registered in the selected UPHC and RPHC of Puducherry during 2019-2022 (N=1127) ^$^Other comorbidity includes bronchial asthma, thyroid, heart disease, epilepsy, stroke, and renal diseases.^¥^Patients receiving treatment from health centres by themselves, or their proxies were considered as on treatment (as per the records/entries made in their treatment cards). RPHC: Rural primary health centre; UPHC: urban primary health centre

Variables	UPHC (n= 622)	RPHC (n=505)
	n (%)	n (%)
Age at the time of registration (years)		
18-40	36 (5.8)	28 (5.5)
41-60	309 (49.7)	276 (54.5)
Above 60	277 (44.5)	201 (39)
Gender		
Male	188(30.2)	208 (41.2)
Female	434 (69.8)	297 (58.8)
Comorbidities		
No comorbidity	277 (44.5)	221 (43.8)
Comorbidity present	345 (55.5)	242 (56.2)
Diabetes	247 (39.6)	202 (40.0)
Diabetes with Other	42 (5.8)	19 (3.8)
Other^$^	56 (9.0)	21 (4.1)
Treatment status		
On treatment from UPHC or RPHC^¥^	506 (81.4)	454 (89.7)

**Figure 3:**
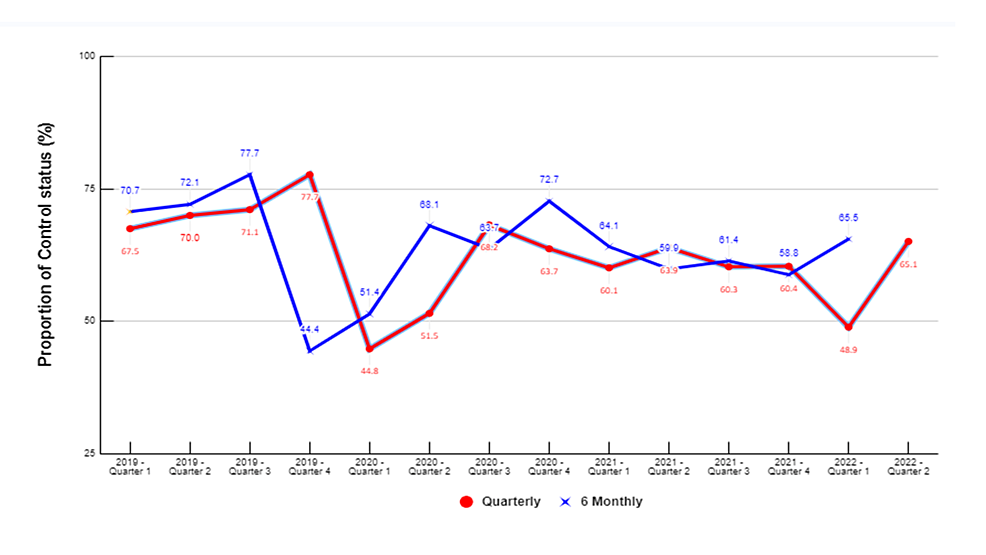
Quarterly and six-monthly control status of hypertension for patients registered at the UPHC from 2019 to 2022 (N=622) UPHC: Urban primary health centre

**Figure 4:**
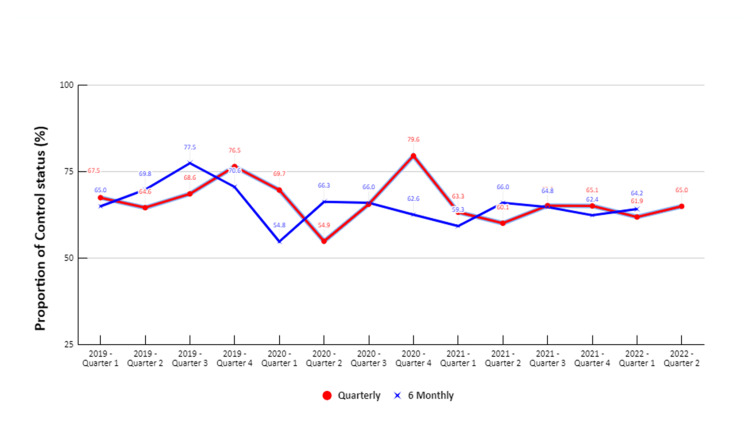
Quarterly and six-monthly control status of hypertension for patients registered at the RPHC from 2019 to 2022 (N=505) RPHC: Rural primary health centre

Figures 3, 4 illustrate the quarterly and six-monthly control levels from 2019 to 2022. The overall quarterly and six-monthly control rates ranged from 45% to 80%. At the UPHC, the highest control rate of 78% was observed after six months of treatment for a cohort in Q-3 of 2019, while the lowest rate of 44.4% was seen after six months of treatment for a cohort in Q-4 of 2019. In the RPHC, the highest control rate of 80% was observed after six months of treatment for the cohort registered in Q-4 of 2020. The lowest control rate of 55% was recorded for cohorts registered in Q-1 and Q-2 of 2020 after three and six months of treatment initiation, respectively.

Table 2 displays the proportion of patients with adequately controlled hypertension, defined as individuals with BP under control for 10 or more months within the year. At UPHC, the proportion ranged from 41% to 46%, while at RPHC, it varied from 33% to 45%.

**Table 2:** Yearly adequate control rate at the UPHC and RPHC from 2019 to 2022 RPHC: Rural primary health centre; UPHC: urban primary health centre

RPHC	Total Registered	Adequately Controlled	UPHC	Total Registered	Adequately Controlled
Year	N	n (%)	Year	N	n (%)
2019	351	157 (44.7)	2019	401	179 (44.6)
2020	398	184 (46.2)	2020	452	193 (42.6)
2021	423	192 (45.3)	2021	556	189 (33.9)
2022	505	209 (41.4)	2022	622	206 (33.1)

In the fourth quarter of 2022, 436 participants with hypertension, who were receiving treatment at NCD clinics in two centres, were randomly selected to investigate the potential association between various socio-demographic or clinical characteristics and hypertension control status. The aim was to understand factors that might influence hypertension management.

The analysis revealed that factors such as age, gender, socioeconomic class, and duration of treatment did not demonstrate any significant association with controlled hypertension during Q-4 of 2022. However, it was observed that participants aged between 41 and 60 years had a 1.67 times higher prevalence of controlled hypertension compared to those aged 18-40 years. The relevant data and results are presented in Table 3.

**Table 3:** Association of socio-demographic factors with hypertension control status in Q-4 of 2022, among patients with hypertension seeking treatment at NCD clinics from October 2022 to January 2023 (N=436) ^€^As per BG Prasad scale, *To ensure sufficient sample size, the upper and upper middle classes were merged as there were fewer participants in both groups. NCD: Non-communicable disease

Variables	n (%)	Controlled hypertension ^# i^n Q-4 of 2022 n (%)	Prevalence Ratio (95% CI)	p-value
Age (years)				
18-40 (ref)	14 (3.2)	6 (42.9)	Ref	-
41-60	188 (43.1)	135 (71.8)	1.67 (0.90-3.08)	0.03
61-80	218 (50.0)	140 (64.2)	1.5 (0.81-2.76)	0.12
>81	16 (3.7)	13 (81.3)	1.89 (0.99-3.62)	0.04
Gender				
Female (ref)	298 (68.3)	200 (67.1)	Ref	-
Male	138 (31.6)	94 (68.1)	1.01 (0.88-1.16)	0.84
Socioeconomic class^€^				
Upper class / Upper middle class (ref)^*^	26 (6.0)	17 (65.4)	Ref	-
Middle class	74 (17)	46 (62.2)	0.95 (0.68-1.32)	0.78
Lower middle class	166 (38.1)	116 (69.9)	1.06 (0.79-1.43)	0.64
Lower class	170 (39)	115 (67.6)	1.03 (0.76-1.39)	0.81
Duration of Treatment				
More than 5 years (ref)	189 (43.3)	121 (64)	Ref	-
0-5 years	247 (56.7)	173 (70)	1.09 (0.95-1.25)	0.18

## Discussion

In the Puducherry district, a record-based cross-sectional study was conducted at selected primary urban and rural health centres to assess the control status of hypertension. The study employed a cohort analysis approach to estimate the control rates. Throughout the study period, the quarterly and six-monthly control rates ranged from 60% to 70% for each quarter evaluated. Notably, there was a disparity in the control status between the two centres. For instance, during quarter 1 of 2022, the six-monthly control rate was 62% at an RPHC and 48% at an UPHC. The indicators used for cohort monitoring were adapted from the WHO HEARTS technical package, which is recommended for use in primary care settings in low-resource settings.

Our study findings align to some extent with the results reported by the IHCI project. According to their findings, the hypertension control status for patients registered in HWCs was 55%, in PHCs was 48%, in hospitals was 44%, and in CHCs was 37% [10]. The improved BP control in our study settings can be attributed to various factors, including the availability of medicines, proximity of healthcare facilities to patients' residences, positive patient and healthworker relationships, and a high doctor-to-patient ratio. It is essential to highlight that our study, conducted in India, is among the first to adopt a cohort monitoring approach as recommended in the HEARTS package. This approach was specifically used to report the longitudinal control rate of hypertension [9]. A study estimated the quarterly control status of 242 individuals with hypertension receiving treatment at an urban health centre. The control status was assessed during two consecutive quarters, Q-1 (January-March) and Q-2 (April-June) of 2022, for individuals who were registered with hypertension in Q-4 (October and December) of 2021. In contrast to our study, where we observed a hypertension control status of 63% during Q-2, this study found a lower control status of 32% in Q-1 and 51% in Q-2 of 2022. Unlike our study, this study specifically examined missed visits as a variable in the quarterly outcomes [11].

Regular monitoring plays a crucial role in yielding valuable information about clinical disease management, program performance, patient outcomes, and the long-term impact of healthcare on both patients and healthcare facilities [12]. One specific aspect of this monitoring is identifying patients who miss clinic appointments for a quarter, which holds significant relevance. This identification allows healthcare providers to target patients and make outreach efforts through means such as telephonic follow-ups, ASHAs, or social workers, encouraging patients to attend clinics to avoid potential complications, ensure uninterrupted drug intake, and promote continuity of care. Moreover, tracking loss to follow-up can serve as an indicator of both the clinic's performance and its acceptability to patients. Understanding why patients may be lost to follow-up provides valuable insights into their attitudes toward accessing healthcare services, either seeking care early or delaying it [13].

During Q-2 of 2020, there was a notable decline in the availability of six-monthly BP readings for patients registered in Q-3 of 2019. This decline was more pronounced in UPHC, where the proportion of unavailable BP readings among patients registered for Q-2 of 2020 reached 97%, while in RPHC, it was 32%. The primary reason behind this decrease can be attributed to the disruption in NCD services caused by the COVID-19 pandemic and the subsequent lockdown response [14]. Due to the pandemic, public NCD screening programs were suspended, impacting the delivery of healthcare services across India. However, NCD services were still considered essential and were meant to be provided despite the challenges posed by a limited workforce. Nevertheless, the COVID-19 pandemic led to a significant 49% disruption in planned training and a 26% delay in implementation exercises in health centres following the WHO-Package of Essential Non-communicable Disease Interventions (WHO-PEN) guidelines [15]. As a result of the pandemic, patient-doctor interactions and visits to PHCs decreased substantially, affecting various aspects of hypertension management. Although anti-hypertensive medications were still dispensed to patients through health workers, the reduced patient-doctor interactions hindered critical activities such as regular BP measurement, dose titration, detailed assessment, and counseling for patients with hypertension [14]. This disruption in regular healthcare services during the pandemic likely contributed to the lower availability of six-monthly BP readings for registered patients.

Our study revealed a noteworthy and statistically significant finding, indicating a decline in hypertension control as individuals age, with a p-value of <0.05. This trend is consistent with numerous previous studies that have also observed a similar association between aging and reduced hypertension control. In addition to age, we investigated several other variables such as social class, occupation, and duration of illness; however, our findings did not demonstrate statistical significance for these factors, despite their relevance in other research [8]. Further research is warranted to show the transition overtime periods as the increase in population longevity, influenced by socioeconomic and demographic shifts, is likely to result in a noteworthy upsurge in the absolute and relative demand for hypertension care in middle-income countries such as India [16,17]. The observed decrease in hypertension control with advancing age underscores the challenges faced by older individuals in managing their BP effectively. As individuals grow older, hypertension management becomes more complex, necessitating tailored interventions and heightened awareness among this specific age group.

The study has several notable strengths that contribute to the robustness of its findings. The data collection process adhered to a uniform protocol, ensuring consistency and minimizing potential biases in the study. Additionally, the use of the most recent BP readings extracted from case sheets, collected by the same investigator, further enhances the reliability and accuracy of the results. A significant strength of the study is the adoption of a cohort analysis approach using a standard methodology which is a novelty of this study. However, the findings are limited to the study population and may not be generalizable to other settings. The study also highlights challenges such as disruptions in NCD services during the COVID-19 pandemic and the high proportion of missing BP readings in patient records may introduce bias and affect conclusive findings.

In future prospects, adopting a prospective approach and employing analytical epidemiological methods to investigate various risk factors of hypertension, such as BMI, salt intake, treatment compliance, and adherence, will yield valuable insights. Additionally, understanding the reasons behind missed visits and potential dropouts from the study could provide a comprehensive understanding of the dynamics influencing hypertension management and contribute to the development of more effective interventions.

## Conclusions

The primary objective of our study was to assess the control status of individuals with hypertension in both urban and rural primary health centers across a specific timeframe. The results demonstrated varying rates of control within these centers. When exploring potential factors influencing control rates, only age exhibited a significant association, while other factors did not show statistical significance.

Our research highlights the critical role of data accessibility, availability, and effective hypertension monitoring systems in enhancing strategies and interventions for hypertension management. The presence of consistent and dependable longitudinal data facilitates evidence-based healthcare approaches, ultimately leading to an enhanced quality of care for individuals with hypertension.

Numerous initiatives have been implemented to ensure regular monitoring and meticulous recording of cohort details for various health conditions. If similar methodological approaches were nationally reinforced, it could substantially decrease the prevalence of associated risk factors and their subsequent complications.
